# Glycerol biosynthetic pathway plays an essential role in proliferation and antioxidative defense in the human enteric protozoan parasite *Entamoeba histolytica*

**DOI:** 10.1038/s41598-023-40670-z

**Published:** 2023-09-05

**Authors:** Ghulam Jeelani, Emmanuel Oluwadare Balogun, Afzal Husain, Tomoyoshi Nozaki

**Affiliations:** 1 Department of Biomedical Chemistry, Graduate School of Medicine, The University of Tokyo, 7-3-1 Hongo, Bunkyo-ku, Tokyo, 113-0033 Japan; 2 Department of Biochemistry, Ahmadu Bello University, Zaria, Nigeria; 3 Department of Biochemistry, Faculty of Life Sciences, Aligarh Muslim University, Aligarh, India

**Keywords:** Parasitology, Parasite biology

## Abstract

Amebiasis is caused by the protozoan parasite *Entamoeba histolytica*. Treatment options other than metronidazole and its derivatives are few, and their low efficacy against asymptomatic cyst carriers, and experimental evidence of resistance in vitro justify the discovery/repurposing campaign for new drugs against amebiasis. Global metabolic responses to oxidative stress and cysteine deprivation by *E. histolytica* revealed glycerol metabolism may represent a rational target for drug development. In this study using ^14^C-labelled glucose, only 11% of the total glucose taken up by *E. histolytica* trophozoites is incorporated to lipids. To better understand the role of glycerol metabolism in this parasite, we focused on characterizing two important enzymes, glycerol kinase (GK) and glycerol 3-phosphate dehydrogenase (G3PDH). Recombinant GK was biochemically characterized in detail, while G3PDH was not due to failure of protein expression and purification. GK revealed novel characteristics and unprecedented kinetic properties in reverse reaction. Gene silencing revealed that GK is essential for optimum growth, whereas G3PDH is not. Gene silencing of *G3PDH* caused upregulated *GK* expression, while that of *GK* resulted in upregulation of antioxidant enzymes as shown by RNA-seq analysis. Although the precise molecular link between GK and the upregulation of antioxidant enzymes was not demonstrated, the observed increase in antioxidant enzyme expression upon GK gene silencing suggests a potential connection between GK and the cellular response to oxidative stress. Together, these results provide the first direct evidence of the biological importance and coordinated regulation of the glycerol metabolic pathways for proliferation and antioxidative defense in *E. histolytica,* justifying the exploitation of these enzymes as future drug targets.

## Introduction

*Entamoeba histolytica* is a protozoan parasite that causes intestinal colitis, dysentery, and extraintestinal abscesses in humans^1^. Globally, approximately 50 million people suffer from invasive amebic infection, resulting in 40–100 thousand deaths annually worldwide^2^. Metronidazole and related 5-nitroimidazole compounds are commonly used against invasive intestinal and extraintestinal amoebiasis^3^. Although clinical resistance against metronidazole has not yet been demonstrated, sporadic cases of treatment failure have been reported^4,5^. In addition, it has been shown that *E. histolytica* is capable of surviving and gaining resistance to sub-therapeutic levels of metronidazole in vitro^6,7^. Therefore, new drugs with different targets or mechanisms of action are needed and thus, to this end, the identification and characterization of metabolic pathways and enzymes that are essential for survival and proliferation of the parasite must be investigated.

**Figure 1:**
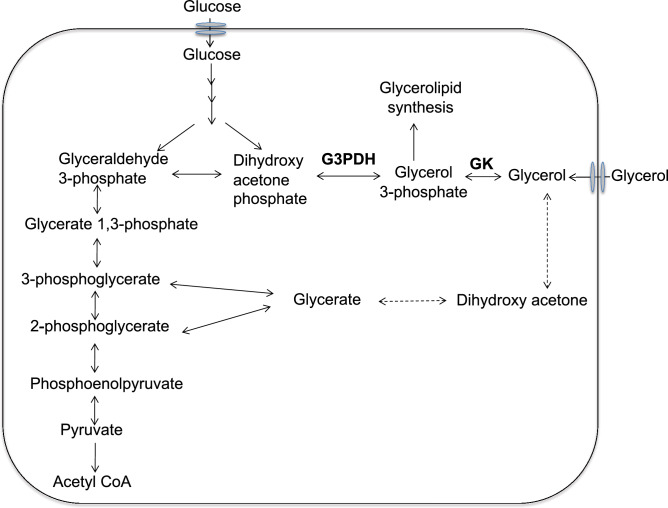
Scheme for predicted glycerol metabolism in *E. histolytica*. Solid lines represent the steps catalyzed by the enzymes whose encoding genes are present in the genome, whereas dashed lines indicate those likely absent in the genome or not identified so far. *G3PDH* glycerol 3-phosphate dehydrogenase, *GK* glycerol kinase.

*E. histolytica* resides in the human colon, which is an anaerobic or microaerophilic environment, and lacks features of aerobic energy metabolism such as TCA cycle, electron transport chain, and oxidative phosphorylation, and relies solely on glycolysis for ATP supply^8,9^. The activity of all glycolytic enzymes was demonstrated in extracts of amoebic trophozoites cultured under axenic or monoxenic conditions^10,11^. In contrast, the presence and significance of glycerol metabolism remains promiscuous in this parasite: glycerol 3-phosphate (G3P) dehydrogenase (G3PDH) activity was not detected in *E. histolytica* trophozoites by conventional enzymatic methods^12^, and dihydroxyacetone phosphate (DHAP) appears to be mainly used for synthesis of triglyceride, but not G3P^12^. In the cell, glycerol is either incorporated from extracellular milieu or synthesized by reduction of DHAP by glycerol dehydrogenase, yielding G3P in the glycolytic pathway (Fig. 1). We previously demonstrated that G3P and glycerol was most highly upregulated among ~ 100 metabolic intermediates under oxidative stress metabolites, strongly suggesting the importance of glycerol metabolism in oxidative stress response and defense^13^. Identification of glycerol 3-phosphate dehydrogenase (G3PDH) and glycerol kinase (GK) in the *E. histolytica* genome (AmoebaDB; https://amoebadb.org/amoeba/app/) ensures the presence of glycerol metabolism in this parasite.

In the present work, we investigated the role of glycerol metabolism in *E. histolytica*. Using metabolomics analysis with ^14^C-labelled glucose, we have shown that only 11% of the total glucose taken up by *E. histolytica* trophozoites is incorporated to lipids, suggesting that G3P at the intersection of glycerol and phospholipid metabolism is primarily used for triglyceride synthesis. Furthermore, biochemical characterization of recombinant GK demonstrated both kinase and phosphatase activities. Reverse genetic studies in which GK expression was silenced by transcriptional gene silencing have demonstrated that repression of GK causes growth defect, suggesting importance of this enzyme for parasite proliferation. We have further shown that gene silencing of *EhG3PDH* causes 30.7 fold upregulation of *EhGK gene*, suggesting the presence of regulatory feedback mechanism in the glycerol metabolic pathway of *E. histolytica.* We have also demonstrated that GK mainly operates in the forward reaction (G3P production) in normal conditions; in contrast, however, under oxidative stress, GK is capable of catalyzing the reverse reaction, leading to formation of glycerol and ATP. Overall, our results have underpinned the important role of glycerol pathway for the parasite survival, thus warranting the exploitation of the pathway and enzymes as potential drug targets.

## Results

### Minor contribution of glucose for glycerolipid biosynthesis in *E. histolytica*

**Figure 2:**
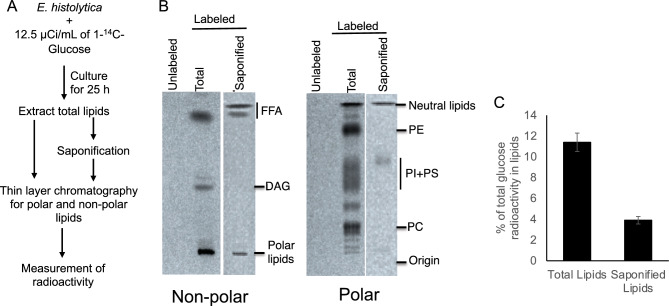
Incorporation of glucose into the *E. histolytica* lipids. (**A**) A schematic flow of the experimental strategy to analyze the incorporation of glucose into lipids. *E. histolytica* trophozoites were cultured in the presence or absence of 12.5 µCi/mL of 14C^1^-glucose for 25 h. Lipids were extracted from cell pellets, either left untreated or subjected to saponification, and analyzed by thin layer chromatography (TLC). (**B)** Thin layer chromatography of 14C^1^-glucose labeled or unlabeled non-polar (left) and polar lipids (right). *FFA* free fatty acid, *DAG* diacylglycerol, *PE* phosphatidylethanolamine, *PI* phosphatidylinositol, *PS* phosphatidylserine, *PC* phosphatidylcholine. The full TLC image is presented in Supplementary Fig. S4. (**C**) Quantitative analysis of the total glucose radioactivity into lipids. Data shown are the means ± standard deviations of three biological replicates.

Since glucose is in general the major starting substance for glycerol-related metabolism, we first examined incorporation of [1-^14^C]-glucose into lipids. Lipids were extracted from trophozoites cultured in either presence or absence of [1-^14^C]-glucose for 25 h and analyzed by high performance thin layer chromatography (Fig. 2A). We found that ^14^C atoms of [1-^14^C]-glucose was incorporated into various polar and non-polar lipids (Fig. 2B). Further quantitative analysis revealed that only 11.4 ± 0.89% of the total glucose taken up by trophozoites was incorporated into lipids (Fig. 2C), suggesting that the majority of glucose was utilized by glycolysis and glycerol metabolism.

### Identification of *G3PDH* and *GK* genes in the genome of *E. histolytica*

We identified genes encoding G3PDH and GK from *E. histolytica* by blastp search against the *E. histolytica* genome database (http://amoebadb.org/amoeba/) using *Escherichia coli* G3PDH (accession number, WP_060643474; E value, 6e−24; % coverage, 89; % identity, 24.5) and GK (GCH09048; 3e−149; 98; 45) sequences as queries. We designated the putative corresponding genes as *EhG3PDH* (EHI_099700 and XM_644519, XP_649611) and *EhGK* (EHI_164850 and XM_650121, XP_655213), respectively. *EhG3PDH* gene contains a 3201 bp open reading frame, which encodes the protein of 1066 amino acids with a predicted molecular mass of 116.2 kDa and p*I* of 6.6, while *EhGK* gene contains a 1458 bp open reading frame, which encodes the protein of 485 amino acids with a predicted molecular mass of 53.7 kDa and p*I* of 5.3.

### Features of EhG3PDH and EhGK

**Figure 3:**
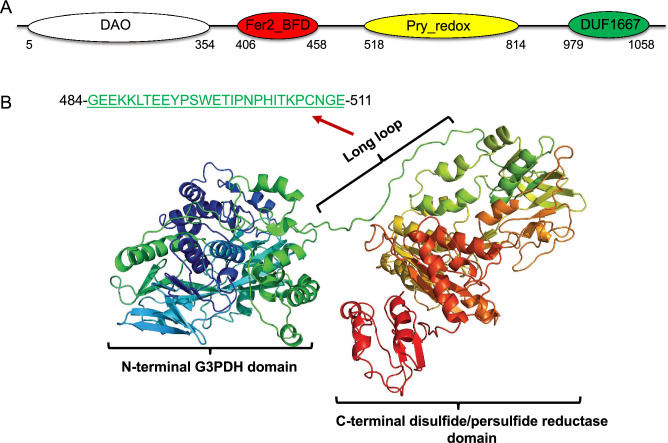
Domain organization and structure modelling of *E. histolytica* G3PDH. (**A**) Domain organization of EhG3PDH. Residues 5-458 correspond to the multidomain TIGR03377 of subunit A (GlpA) protein family of FAD-dependent anaerobic glycerol-3-phosphate dehydrogenase. Abbreviations are: FAD-dependent oxidoreductase domain (DAO, Pfam PF01266, residues 5-354); bacterioferritin-associated ferredoxin (BFD)-like [2Fe-2S] binding domain (Fer2_BDF; PF04324, residue 406-458); pyridine nucleotide-disulphide oxidoreductase family that includes a small NADH binding domain within a larger FAD binding domain (Pyr_redox_2, PF07992, residues 518-814); and a domain found in hypothetical metal-binding proteins (DUF 1667, residues 979-1058). (**B**) Predicted structure of EhG3PDH. The protein likely comprises two functional structural domains, the N-terminal G3PDH and the C-terminal disulfide/persulfide reductase. The two domains are linked by a long flexible loop (484-511).

To identify conserved functional domains in the protein sequence, we employed NCBI's conserved domain (https://www.ncbi.nlm.nih.gov/Structure/cdd/wrpsb.cgi) and Eukaryotic Linear Motif (ELM) prediction tools (http://elm.eu.org/). EhG3PDH reveals unique domain organization and possesses four domains: DAO (D-amino acid oxidase) [FAD-dependent oxidoreductase domain; Pfam PF01266) domain, bacterioferritin-associated ferredoxin (BFD)-like [2Fe-2S]-binding (Fer2_BFD) domain, small NADH binding domain within a larger FAD binding domain (Pyr_redox_2, PF07992), and DUF 1667 domain (Fig. 3A). The DAO domain is found in various FAD-dependent oxidoreductases such as G3PDH and D-alanine oxidase. The BFD-like [2Fe-2S] domain corresponds to bacterioferritin-associated ferredoxin like [2Fe-2S]-binding domain of anaerobic G3PDH subunit A. The BFD-like [2Fe-2S]-binding domain is also found in a variety of proteins including BFD, which is the large subunit of NADH-dependent nitrite reductase. The third domain is commonly found in the pyridine nucleotide-disulphide oxidoreductase family flavoproteins (e.g., thioredoxin reductases (TRxR), NADH oxidases, and peroxidases). The last domain (DUF 1667) was previously found in archaeal and bacterial hypothetical proteins, some of which are annotated as potential metal-binding proteins.

Further database search using EhG3PDH as query showed that only two protozoan parasites of the genus *Giardia* (*G. lamblia* (XP_001707988) and diplomonad *Spironucleus salmonicida* (XP_649611) encode a single protein that appears to be orthologous to EhG3PDH in the domain architecture. Overall, EhG3PDH of 1066 amino acids habours two functional domains of approximately ~ 500 amino acids each of the glycerophosphate dehydrogenase domain (Supplementary Fig. S2A) at the N-terminus and the disulfide/persulfide reductase domain (Supplementary Fig. S2B) at the C-terminus. These two domains are separated by a 28-a.a. long linker region of (a.a. 484-511) (Fig. 3B). The structure of EhG3PDH predicted by Phyre2^14^ using closely-related structures as templates suggests that the linker loop is likely hyperflexible, well separates the G3PDH and disulfide/persulfide reductase domains, and may mediate interaction of the two domains by structural changes (Fig. 3B). The Phyre2 analysis was conducted in the "intensive" mode, with > 98% of the amino acid residues modeled at > 90% confidence, ensuring the robustness of the predictions. The predicted structure supports the presence of a flexible linker domain between the N-terminal G3PDH and C-terminal disulfide/persulfide reductase domains. This linker is essential for the coordinated functionality of both domains, considering the overall size of the protein and the size of the fused domains.

**Figure 4:**
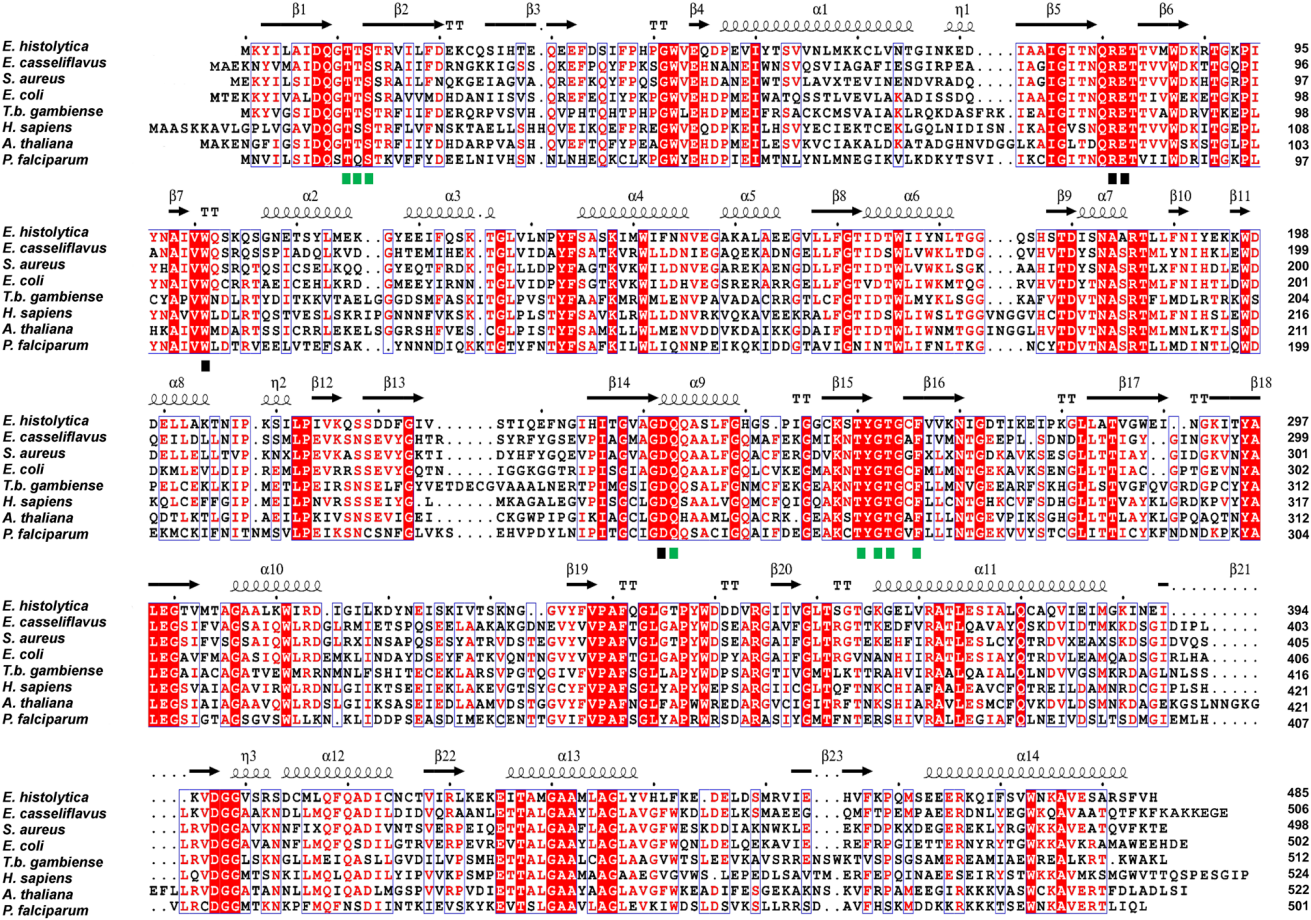
Protein sequence alignment of GKs. The alignment was created with ESPript 3.0 alignment editor^16^. The species names of organisms shown as abbreviations and their NCBI accession numbers are: *Enterococcus casseliflavus* (OQO84716), *Staphylococcus aureus* (OHS88669), *Escherichia coli* (KIG63106)*, T. b. gambiense* (BAI79241), *Homo sapiens* (NP_000158), *Arabidopsis thaliana* (AAO61418)*, and Plasmodium falciparum* (CAD52637). The predicted secondary structure elements identified in EhGK are shown above the alignment: arrows (β-strands), TT (β-turns), coils (α-helices), and η (3_10_-helix). Amino acid residues shown in white with a red background shade are the residues conserved among GKs. Amino acid residues implicated for the substrate binding in *T. b. gambiense* GK are indicated by rectangles beneath the alignment (green rectangles, ADP; black rectangles, catalytic glycerol).

In contrast to EhG3PDH, EhGK apparently has a simple domain structure with no apparent deletion or insertion and revealed up to 42% identity to GK from other organisms at the protein level (Fig. 4). Similar to GKs and other members of the sugar kinase families, EhGK is predicted to possess core contiguous multiple β-sheets, which contribute to overall secondary structure arrangements similar to *Trypanosoma brucei* GK^15^. Comparison of EhGK against the primary and secondary structures of *T. brucei* GK identified the amino acid residues that are likely involved in the coordination of substrates in EhGK (T11, T12, S13, Q243, T260, G262, T263, F266 for ADP/ATP binding; and Q80, E82, W101, D242 for glycerol/G3P binding).

### Kinetic properties of rEhGK

**Figure 5:**
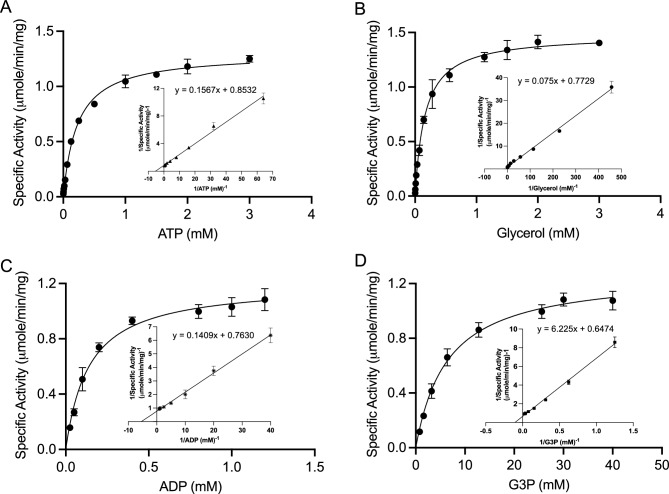
Kinetic studies for *E. histolytica* GK. Specific activity data for all substrates in the forward and reverse directions were fitted to Michaelis–Menten equation and transformed to Lineweaver–Burk plots (inserted panels), which were used for the determination of the kinetic parameters for (**A**) ATP, (**B**) glycerol, (**C**) ADP and (**D**) glycerol 3-phosphate. All measurements were done in triplicates and presented as mean ± SEM.

The enzymatic characteristics of EhGK were determined. The purified recombinant EhGK was highly active in both the forward (ATP and glycerol as substrate) and reverse direction (ADP and G3P as substrate). The requirement of metal ions was examined in the forward (G3P forming) reaction: EhGK showed an absolute requirement for a free bivalent metal as cofactors, with Mg^2+^ as the preferred cation (Table 1). No activity was detected in the presence of monovalent metal ions (Li^+^, Na^+^, and K^+^). The enzyme displayed a broad and symmetrical profile, showing an activity of 50–100% over a wide range of pH (6.0–7.5) measured at 37 °C, with the optimum (100% activity) being pH 7.5. Therefore, all kinetic studies were performed at pH 7.5. The kinetic constants, *K*_m_ and *V*_max_ were determined by measuring the initial velocities of EhGK reaction in both the forward and reverse reactions over a wide range of substrate concentrations (Fig. 5A–D, Table 2).

**Table 1:** Effects of metal ions on the activity of EhGK^a^.

Metal^b^	Relative activity (%)
None	ND
MgCl_2_	100 ± 5
FeCl_2_	78 ± 7
CaCl_2_	45 ± 5
CoCl_2_	25 ± 6
MnCl_2_	16 ± 4
ZnCl_2_	13 ± 2
NiCl_2_	5 ± 1
CuCl_2_	2 ± 1
LiCl	ND
NaCl	ND
KCl	ND

^a^Assays were performed as described in “Materials and methods” section, in the presence of 100 mM Tris–HCl, pH 7.5, 1 mM glycerol, and 1 mM ATP.

^b^The concentration of cations used was 5 mM. The activity is shown in percentage (%) relative to that measured in the presence of MgCl_2_. *ND* not detected.

**Table 2:** Kinetic parameters of EhGK in forward (ATP- and glycerol-utilizing) and reverse (ADP- and G3P-utilizing) directions.

Substrate	*K*_m_	*V*_max_
	(mM)	(µmol min^−1^ mg^−1^)
ATP	0.23 ± 0.03	1.30 ± 0.04
Glycerol	0.16 ± 0.03	1.48 ± 0.05
ADP	0.14 ± 0.04	1.20 ± 0.08
G3P	8.71 ± 2.46	1.28 ± 0.14

GK forward and reverse activities were assayed as described in “Materials and methods” section with different concentrations of ATP (0.01–3 mM) and glycerol (0.002–3 mM) or G3P (0.8–40 mM) and ADP (0.025–1.2 mM). Values are expressed as mean ± S.D. of three independent experiments.

### Intracellular distribution of EhG3PDH and EhGK

To examine intracellular distribution of EhG3PDH and EhGK in trophozoites, we established transformant lines expressing EhG3PDH and EhGK fused to the hemagglutinin (HA) tag at the amino terminus (HA-EhG3PDH and HA-EhGK). The expression of these proteins in trophozoites was confirmed by immunoblot analysis with anti-HA antibody (Supplementary Fig. S3). We first examined the distribution of EhG3PDH and EhGK in trophozoites by immunoblotting using fractions of lysates that had been produced by mechanical homogenization with a Dounce glass homogenizer, followed by centrifugation at 5000×*g* and subsequently at 100,000×*g*. We found that EhG3PDH and EhGK were mainly found in the soluble fraction of centrifugation at 100,000×g, together with a cytosolic soluble protein CS1^17^ (Supplementary Fig. S3). In addition, EhG3PDH and EhGK were also associated with the pellet fraction of centrifugation at 100,000*g*, together with a vesicular membrane protein CPBF1^18^ (Supplementary Fig. S3). These data indicate that EhG3PDH and EhGK were mainly present in the cytosol, but partially localized to the some organelle(s) or particulate compartments.

### Effects of *EhG3PDH *and *EhGK* gene silencing on growth and the cellular glycerol level

**Figure 6:**
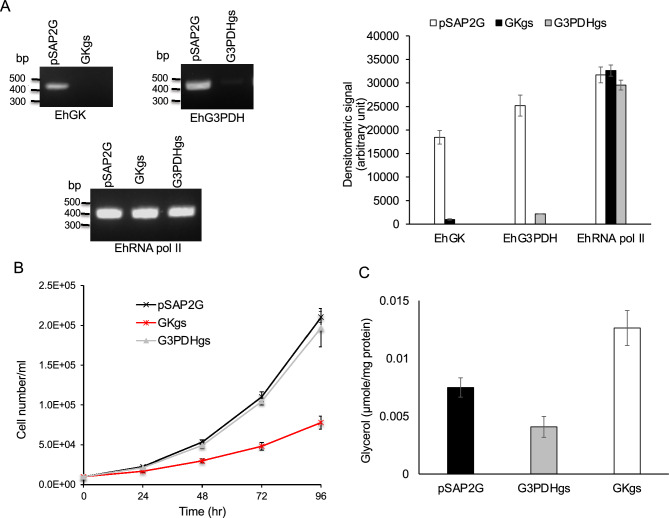
Effects of *EhG3PDH and EhGK* gene silencing on the growth and glycerol levels in *E. histolytica* trophozoites. (**A**) Evaluation of gene expression by semi-quantitative RT-PCR in *EhG3PDH and EhGK* gene silenced strains. The steady-state levels of transcripts of *EhG3PDH, EhGK* and *EhRNA pol II* genes were measured in trophozoites of the strains transfected with mock empty vector (pSAP2G), *EhG3PDH* gene silencing plasmid (pSAP2G-G3PDH), or *EhGK* gene silencing plasmid (pSAP2G-GK). cDNA from the generated cell lines (pSAP2G, G3PDHgs, and GKgs strains) was subjected to 30 cycles of PCR using specific primers for *G3PDH, GK* and *RNA pol II* genes. RNA polymerase II served as a control. PCR analysis of samples without reverse transcription was used to exclude the possibility of genomic DNA contamination. The densitometric quantification of the bands, shown in the right graph, was performed by Image J software (https://imagej.nih.gov/ij/index.html), and the expression level of *EhG3PDH, EhGK*, and *EhRNA pol II* was expressed in arbitrary units. Data shown are the means ± standard deviations of three biological replicates. The full-length gel image is shown in Supplementary Fig. S5. (**B**) Growth kinetics of control (pSAP2G), *EhG3PDH* gene silenced (G3PDHgs) and *EhGK* gene silenced (EhGKgs) transformants. Approximately 6000 amoebae in the logarithmic growth phase were inoculated into 6 mL fresh culture medium and amoebae were then counted every 24 h. Data shown are the means ± standard deviations of five biological replicates. (**C**) Intracellular amounts of glycerol in pSAP2G, G3PDHgs and EhGKgs transformants. The data are presented as mean ± SD of glycerol in µmol per mg protein. All measurements were done in triplicates.

To investigate the physiological importance of G3PDH and GK, we created and examined an *E. histolytica* strain where *EhG3PDH* or *EhGK* gene was silenced by antisense small RNA-mediated transcriptional gene silencing^19^. RT-PCR followed by agarose gel electrophoresis confirmed repression of *EhG3PDH* and *EhGK* gene expression (Fig. 6A). We examined the growth kinetics of trophozoites of *EhG3PDH* and *EhGK* gene-silenced and mock pSAP2G-transfected control strains in regular BI-S-33 medium (Fig. 6B). We found that *GK* gene silenced transformants displayed a severe growth defect (the population doubling time, 31.8 ± 0.80 h), whereas *G3PDH* gene-disrupted transformants showed no growth defect (21.6 ± 0.54 h) (Fig. 6B), when compared to mock control strain (20.7 ± 0.54 h). These results indicate that GK is involved in proliferation and needed for optimum growth in vitro.

We then compared the level of glycerol in the gene silenced and control transformant strains. The glycerol level was reduced by 45.5 ± 2.8% reduction in *G3PDH* gene silenced strain as compared to control (Fig. 6C). In contrast, the glycerol level in *GK* gene silenced strain increased by 40.7 ± 2.2% as compared to control (Fig. 6C).

### Effects of *G3PDH *and *GK* gene silencing on the defense against oxidative stress

**Figure 7:**
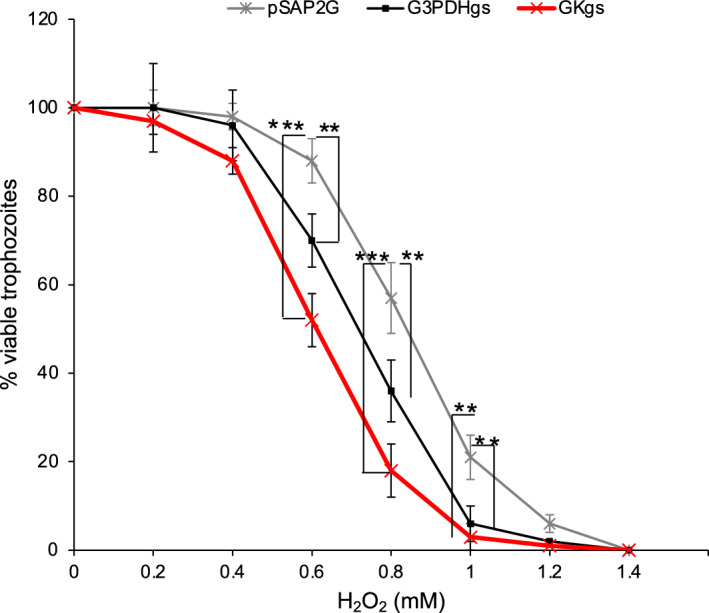
Effect of *EhG3PDH* and *EhGK* gene silencing on oxidative stress tolerance. Trophozoites of G3PDHgs, GKgs and control (harboring plasmid pSAP2G) strains were exposed to different concentrations of H_2_O_2_ for 1 h and viability was determined. Survival rates are shown as the percentage of untreated control cells (mean ± S.D. of triplicates in three independent experiments). Statistical comparisons were made by the *Student*’s *t* test (**P < 0.01; ***P < 0.001).

We also checked if repression of G3PDH and GK also affects the tolerance of trophozoites to H_2_O_2_ mediated cytotoxicity. *EhG3PDH* and *EhGK* gene-silenced and mock control strains were exposed to different concentrations of H_2_O_2_ (0–6.4 mM) for 1 h and viability was evaluated. *EhG3PDH* and *EhGK*, to a larger extent, gene-silenced strains showed slightly, but significantly, higher sensitivity to 0.6–1.2 mM H_2_O_2_ compared to the control transformant, suggesting that G3PDH and GK may be involved in protecting the cells against oxidative stress (Fig. 7). Further investigations are required to elucidate the precise roles of G3PDH and GK in the cellular response to oxidative stress in this parasite.

### Effect of *EhG3PDH* and *EhGK* gene silencing on global transcriptome

Global gene expression analysis was performed using RNA-seq on *EhG3PDH* and *EhGK* gene-silenced strains, as well as mock control strains, to investigate the effects of gene silencing (Supplementary Dataset 1). The rationale behind this analysis was to understand how gene silencing of *EhG3PDH* and *EhGK*, which primarily have metabolic roles, can indirectly globally affect gene expression through the perturbation of cellular metabolism and associated signaling pathways. We also anticipated to identify the up-regulation of genes which presumably have genetic interaction(s) with the silenced gene, by compensatory mechanisms which assist the parasite to survive.

The genes whose transcripts were up- or down-regulated more than threefold (with the q-value < 0.005) in each gene silenced strain compared to control, are shown in Supplementary Dataset 2. In *EhG3PDH* gene-silenced strain, 137 genes were down-regulated (Supplementary Dataset 2) and 55 genes were up-regulated (Supplementary Dataset 2). The *EhG3PDH* transcript level was reduced by 160.6 fold (Supplementary Dataset 2). Among the genes that were significantly down-regulated, those encoding for several hypothetical proteins (EHI_144490, EHI_114950, EHI_154310, EHI_035100, EHI_156220), several AIG1 family proteins (EHI_041465, EHI_176590, EHI_176700, EHI_180390, EHI_176580) which plays a pivotal role in *E. histolytica* virulence via regulation of host cell adhesion^20^. 40S ribosomal protein S14 (EHI_074090), amino acid transporter (EHI_190460), phospholipid-translocating P-type ATPase (EHI_174280), calmodulin (EHI_117470), phosphatidate cytidylyltransferase (EHI_163240), which participates in glycerophospholipid metabolism and phosphatidylinositol signaling system^21^ (Supplementary Dataset 2). In contrast, nonpathogenic pore-forming peptide precursor (EHI_169350), which may belong in the saposin-like protein31 (SAPLIP1) family^22^, Lipase 3 (EHI_032470), peroxiredoxin (EHI_139570), Rab family GTPase (EHI_187090), elongation factor 2 (EHI_166810, EHI_164510), aldehyde-alcohol dehydrogenase 2 (EHI_160940) and others genes were upregulated (Supplementary Dataset 2). Interestingly we found that glycerol kinase (EHI_164850) which is related to glycerol pathway was up-regulated by 30.7 fold (Supplementary Dataset 2), suggesting that the increased expression of GK may compensate for the impairment of the G3PDH enzyme.

In *EhGK* gene-silenced strain, 69 genes were down-regulated (Supplementary Dataset 2) and 58 genes were up-regulated (Supplementary Dataset 2), compared to the control strain. The *GK* transcript level was reduced by 3.1 fold (Supplementary Dataset 2), whereas the *G3PDH* transcript level remained unchanged. Similarly to EhG3PDH gene silencing, several hypothetical proteins (EHI_144490, EHI_154310, EHI_114950), AIG1 family proteins (EHI_041465, EHI_176590, EHI_176700, EHI_180390, EHI_176580), 40S ribosomal protein S14 (EHI_074090), amino acid transporter (EHI_190460), phospholipid-translocating P-type ATPase (EHI_174280), calmodulin (EHI_117470), were significantly down-regulated suggesting, involvement of these gene related to glycerol biosynthetic pathway. Gene encoding CDP-alcohol phosphatidyltransferase (EHI_029500) which is involved in phospholipid biosynthesis was down-regulated more than threefold (Supplementary Dataset 2). In contrast, among the most highly upregulated genes was nonpathogenic pore forming peptide (EHI_169350), pyruvate phosphate dikinase (EHI_045080) a key enzyme in gluconeogenesis that is responsible for reversing the reaction performed by pyruvate kinase in Embden-Meyerhof-Parnas glycolysis, several NADPH-dependent FMN reductase domain-containing protein (EHI_067720, EHI_022600,EHI_103260, EHI_181710), and several peroxiredoxin genes (Supplementary Dataset 2).

Surprisingly, we identified a set of genes (41 down regulated and 25 upregulated) that were commonly affected in the same directions (i.e., up or down) in both *EhG3PDH* and *EhGK* gene silenced strains (Supplementary Dataset 3). This observation suggests that these genes may play specific roles in the regulatory pathways associated with glycerol metabolism and glycerolipid synthesis. The shared genes between the two gene silencing experiments could indicate their involvement in core metabolic processes or signaling cascades that are critical for the proper utilization of glycerol and glycerolipid synthesis. The genes in this list may be key regulators or downstream effectors in the pathways affected by the core glycerol metabolism. Further investigations is warranted to determine the exact functions and interplay of these common genes in relation to glycerol metabolism. Their specific roles could shed light on the molecular mechanisms underlying the observed phenotypic differences between *EhG3PDH* and *EhGK* gene silencing and provide a deeper understanding of the complex regulatory networks involved in glycerolipid synthesis in *Entamoeba.*

We also conducted a thorough bioinformatics analysis to further explore the differentially expressed genes and their functional implications in our study. To achieve this, we utilized the g:Profiler online server (http://biit.cs.ut.ee/gprofiler/). Using a cut-off threshold of fold change ≥ 3 and *P* value ≤ 0.05, we identified significantly enriched Gene Ontology (GO) terms for the upregulated and downregulated genes. In the G3PDHgs strain, we observed 2 significantly enriched GO terms among the 137 downregulated genes (Supplementary Dataset 2): "Purine ribonucleotide triphosphate binding" (GO:0035639) and "protein phosphorylation" (GO:0006468). Furthermore, among the 55 upregulated genes (Supplementary Dataset 2), we identified 2 significantly enriched GO terms: "GTP binding" (GO:0005525) and "actin filament" (GO:0051015). For the EhGKgs strain, we found one enriched GO term among the 69 downregulated genes (Supplementary Dataset 2): "GTP binding" (GO:0005525). Additionally, among the 58 upregulated genes (Supplementary Dataset 2), only one significantly enriched GO term was observed: "oxidoreductase activity" (GO:0016491). The identification of these enriched GO terms offers important insights into the biological processes and molecular functions affected by the gene silencing in the respective strains. These findings shed light on potential pathways and functions that may be regulated by the altered expression of the target genes.

## Discussion

### Importance of glycerol and lipid metabolism for *E. histolytica* pathogenesis

Trophozoites of *E. histolytica* exhibit dynamic membrane-associated activities such as motility, membrane traffic, phago- and trogocytosis, all of which are essential for pathogenesis. Lipids are centrally involved in all the above mentioned processes, and therefore molecular dissection and elucidation of lipid metabolism is important to understand pathophysiology of the parasite. As all lipids have a glycerol backbone and glycerophospholipids biosynthesis is initiated when two fatty acids are transferred to G3P, G3P is positioned at the gateway of lipid and carbohydrate metabolism^23^. Furthermore, G3P serves at a critical node in the control of triacylglycerol synthesis in mammals^24^. In the absence of external sources, glycerol is synthesized de novo from glucose via the glycolytic pathway (Fig. 1). We demonstrated by metabolic labelling that only 11% of glucose is incorporated into lipids in *E. histolytica* (Fig. 2), suggesting that the parasite utilizes only a small portion of glucose to synthesize the lipids, and glucose is mainly used for energy generation. Our findings indicate that EhGK gene silencing resulted in a growth defect, while EhG3PDH gene silencing did not affect growth. Our data also indicates that the parasite is capable of incorporating glycerol and utilizing it for glycerolipid synthesis. These data suggest that the utilization of glycerol for glycerolipid synthesis is crucial for optimal growth, at least in vitro culture conditions (Fig. 6). Furthermore, *EhGK* gene silencing caused a significant increase in glycerol levels (Fig. 6C). In EhGKgs strain, it is plausible that glycerol incorporated by the parasite cannot be utilized for glycerolipid synthesis, leading to growth retardation (Fig. 6B). On the other hand, in EhG3PDHgs strain, we observed an compensatory upregulation of *EhGK* gene, which in turn caused a reduction in glycerol levels (Fig. 6C). This reduction in glycerol levels in EhGKgs strain suggests that this parasite utilizes glycerol for the synthesis of glycolipids, ultimately maintaining amoeba growth (Fig. 6B). Although glycerol uptake was not demonstrated in the present study, it was previously reported in the malaria parasite *Plasmodium falciparum* that glycerol is taken up from the host serum and used for lipid synthesis and aquaglycerolporin (PfAQP) is involved in the glycerol transport^25^. Furthermore, metabolic labelling demonstrated that erythrocytic stage parasites of *P. knowlesi* are capable of internalizing glycerol and incorporating it into phospholipids^26^. Our genome-wide survey indicated that the *E. histolytica* genome encodes two aquaporin-like proteins (EHI_010520, EHI_195000), which are annotated as hypothetical proteins. It is conceivable that one or both of the genes are involved in glycerol internalization in *E. histolytica*, although their role remains to be demonstrated in future.

### The domain architecture, origin, and role of G3PDH

Glycolysis and fermentation is the major ATP-generating metabolic pathway in *E. histolytica* as this parasite*,* like *G. lamblia* and *T. vaginalis*, lacks all features of aerobic metabolism, including the TCA cycle, the electron transport chain, and oxidative phosphorylation, and generates energy exclusively by substrate level phosphorylation and fermentation^27,28^. G3PDH is a key enzyme at the crossroad of glycolysis, redox, and fatty acid metabolism, in both prokaryotes and eukaryotes^29^. We have shown that the multiple domain architecture of EhG3PDH is unique and resembles only a few organisms including the diplomonad *S. salmonicida* and *G. lamblia*. The presence of orthologs with the same domain configuration (the DAO, Fer2_BFD, and Pyr_redox domains) in the gram-positive anaerobic actinobacteria *Eggerthella* sp. suggests that a first gene fusion event between a GlpA-like G3PDH and a pyridine nucleotide- disulphide oxidoreductase occurred in this or its related prokaryotic lineage. A lateral gene transfer (LGT) between *Eggerthella* sp. or its related organism and the protists, *Entamoeba* sp., *G. lamblia,* and *S. salmonicida*, may account for the shared presence of the orthologues in these organisms. It is worth noting that they apparently share the mammalian intestine as an ecological niche to live. In *G. lamblia,* it has been demonstrated that G3PDH (gG3PD) can convert G3P into DHAP and during encystation gG3PDH activity and the intracellular glycerol level increase^30^. Furthermore, the intracellular localization of gG3PDH is stage dependent and it was partially distributed to mitosomes during encystation. However, our previous transcriptomic analysis on *E. invadens* in a course of encystation^31^ showed that the *EhG3PDH* transcript level decreases more than 13-fold during encystation, while the *EhGK* transcript level increases more than threefold. These data contradict the premise that EhG3PDH is involved in encystation per se in *Entamoeba*, but rather suggests that EhGK may be involved in stage conversion.

### Cross talk of gene network of glycerol metabolism

We also investigated genetic interaction associated with glycerol metabolism by RNA seq analysis of *EhG3PDH* and *EhGK* gene silenced strains. *EhG3PDH* gene silencing caused upregulation of *EhGK* gene expression, which is believed to compensate for the decrease in G3PDH activity and provide additional G3P from glycerol (Supplementary Dataset 2). We found that in *EhG3PDH* gene-silenced strain one of genes encoding lipases (EHI_032470) (Supplementary Dataset 2), which hydrolyze long-chain acyl-triglycerides into di- and monoglycerides, glycerol, and free fatty acids at a water/lipid interface, was up-regulated more than eightfold, suggesting genetic interaction between *EhG3PDH* and this lipase in glycerol metabolism. It has been reported that *Leishmania donovani* promastigotes release lipase activity into the culture milieu, and the activity in turn helps to salvage fatty acids from lipids abundant in both their insect vector and mammalian hosts. The fatty acids may be subsequently used to synthesize complex lipids required for the growth and development of the parasite or used as potential carbon sources in energy metabolism via β-oxidation^32^.

We also found that in *EhG3PDH* gene silenced strain one of the crucial enzymes in the fermentation pathway, aldehyde-alcohol dehydrogenase 2 (EhADH2 or EhADHE, EHI_160940), was > threefold upregulated (Supplementary Dataset 2). EhADH2 is a bifunctional enzyme with both aldehyde dehydrogenase and alcohol dehydrogenase activities, both of which are depended on NADH^33^. It is thus conceivable that upregulation of *EhADH2* gene expression may benefit the parasite to maintain the NADH/NAD^+^ ratios. In *A. thaliana*, similarly, cytosolic G3PDH, not GK, is involved in modulating the NADH/NAD^+^ ratio and the disruption of the encoding gene led to increased NADH/NAD^+^ ratios under standard growth conditions^34^.

In contrast, in *EhGK* gene-silenced strain pyruvate phosphate dikinase (PPDK) was upregulated 46 fold (Supplementary Dataset 2). PPDK, which catalyzes the interconversion of pyruvate to phosphoenolpyruvate, is a key enzyme in gluconeogenesis, while in bacteria and parasites, however, PPDK functions in the direction of ATP synthesis^35^. It has been recently reported in *Entamoeba* and *Giardia intestinalis* that PPDK is involved in the synthesis of ATP. However, the metabolic role of PPDK remains unclear since it has been demonstrated under physiological conditions that the synthesis of ATP is not thermodynamically favorable in *E. histolytica* trophozoites^36^. In a purple nonsulfur anoxygenic photosynthetic bacterium, *Rhodopseudomonas palustris,* PPDK is differentially regulated under different growth conditions and likely play a regulatory role in lipid biogenesis^37^. In *L. mexicana,* it has been suggested that PPDK plays an important role in maintaining the glycosomal energy balance under glucose starvation^38^. We also found that several genes encoding NADPH-dependent FMN reductase domain-containing proteins, which are also known as iron-sulfur flavoproteins (ISFs) and commonly found in anaerobic prokaryotes, were highly upregulated in *EhGK* gene-silenced strain (Supplementary Dataset 2). To date, the only two eukaryotes have been found to possess ISF homologs: *E. histolytica* and *T. vaginalis*. These *ISF* genes were previously shown to be upregulated in *E. histolytica* under the nutrient-deprived conditions^39^.

Surprisingly, in our study, we observed a downregulation (3.6 fold) of gene expression of glyceraldehyde-3-phosphate dehydrogenase (GAPDH), one of the key enzymes involved in glycolysis, in *EhGK* gene-silenced strain (Supplementary Dataset 2), suggesting a potential link between GK and GAPDH. The observed downregulation of *EhGAPDH* in EhGKgs strain highlights the intricate connections between glycerol metabolism, glycolysis, and the regulatory networks involved. Further investigations are warranted to elucidate the precise mechanisms and signaling pathways that underlie the observed relationship between *EhGK* silencing and *EhGAPDH* downregulation.

Taken together, our transcriptomic analyses of *EhG3PDH* and *EhGK* gene silenced strains have shown that interference of glycerol metabolism causes alterations in the expression of genes involved in lipid and carbohydrate metabolism, as well as other pathways in which gene functions are often unassigned. Investigation into the mechanisms of these alterations may provide further insights into the role of glycerol metabolism in this parasite.

### Enzymatic characteristics and the physiological role of EhGK

In general, GKs catalyzes the transphosphorylation of glycerol at the expense of ATP, producing G3P (in the forward reaction)^40^. We have demonstrated in this study that EhGK apparently possesses comparable forward and reverse activities, the latter converts G3P to produce ATP and glycerol. So far, this reverse activity has only been reported for GK from *Trypanosoma brucei*, a group of parasites which cause sleeping sickness and nagana in sub-Saharan Africa^15,41^. Our kinetic studies showed that the kinetic constants such as the Vmax and the Km values in the forward and reverse reactions are comparable except that the Km value for G3P is 54 fold higher compared to that for glycerol (Supplementary Dataset 2). Thus, considering the intracellular concentrations of glycerol and G3P in general^13^, the forward reaction dominates, and the reverse reaction is expected to operate only at high G3P concentrations. Under stress and nutrient-restricted conditions, G3P is accumulated, which is undesirable because G3P is potentially harmful to cells^42^. In *E. histolytica*, it was shown that under oxidative stress conditions, the glycolytic flux was reduced with a concomitant marked accumulation of glycerol^13^. This increase in glycerol was initially presumed to be attributable to dephosphorylation of G3P. The demonstration of the reverse activity of EhGK and the growth defect by gene knockdown of *EhGK* gene (Fig. 6B) have confirmed that EhGK is responsible for the conversion of accumulated G3P to glycerol with a concomitant ATP production for the amoebas to survive under oxidative stress (Fig. 7). This scenario is similar to the case in *T. brucei,* where the parasites were rescued by the reverse activity of GK, which furnished the parasites with ATP for survival, under anaerobic conditions or in the presence of drugs that abolish aerobic glycolysis^43,44^. Tertiary structure of *E. histolytica* GK needs to be demonstrated in future to elucidate the observed preference of the substrates and also to further exploit the enzyme for structure-based drug design.

In summary, we have demonstrated in the present study that *E. histolytica* trophozoites mainly relies on glycerol, but not glucose, for phospholipid synthesis. We have also shown that EhGK is unique in its capability, similar to *Trypanosoma,* of catalyzing the reverse reaction, leading to formation of ATP. Gene silencing of *EhGK,* but not *EhG3PDH,* drastically reduced parasite proliferation, also suggesting that glycerol is an important major source of G3P. Overall, our results have underpinned the role of glycerol metabolism in the parasite survival and have justified exploitation of GK as a potential drug target.

## Materials and methods

### Chemicals and reagents

Glycerol, resazurin, NADP^+^, ATP were purchased from Sigma–Aldrich (Tokyo, Japan). Ni^2+^-NTA agarose was purchased from Novagen (Darmstadt, Germany). 14C^1^-glucose were purchased from Cambridge Isotopes Laboratories (Woburn, MA). Lipofectamine and geneticin (G418) were purchased from Invitrogen (Carlsbad, CA, USA). *Escherichia coli* BL21 (DE3) strain was purchased from Invitrogen. All other chemicals of analytical grade were purchased from Wako (Tokyo, Japan) unless otherwise stated.

### Microorganisms and cultivation

Trophozoites of the *E. histolytica* clonal strain HM1: IMSS cl 6^45^ and G3 strain^19^ kindly provided by David Mirelman, Weisman Institute, Israel, were maintained axenically in Diamond’s BI-S-33 medium at 35.5 °C as described previously^46^. Trophozoites were harvested in the late-logarithmic growth phase for 2–3 days after inoculation of one-thirtieth to one-twelfth of the total culture volume. After the cultures were chilled on ice for 5 min, trophozoites were collected by centrifugation at 500×*g* for 10 min at 4 °C and washed twice with ice-cold PBS, pH 7.4.

### Radioisotope glucose labelling

*E. histolytica* trophozoites were cultured either in absence or presence of 12.5 µCi/mL of 14C^1^-glucose medium for 25 h. Cells were collected and washed with ice cold-PBS, and total lipids were extracted by the Bligh and Dyer’s method^47^. The extracted lipids were analyzed by high performance thin layer chromatography using a solvent system of chloroform:methanol:acetic acid (13:5:2 v/v/v) for separation of polar lipids or Hexane:diethyl ether:acetic acid (7:3:0.1 v/v/v) for non-polar lipids. In order to estimate the incorporation of glucose in glycerol backbone, extracted lipids were subjected to saponification, and analysed on high performance thin layer chromatography as described above. The spots on TLC plates were quantified using a Fuji imaging analyzer.

### Alignment of G3PDH and GK protein sequences

Amino acid sequences of G3PDH and GK like proteins were obtained from other organisms deposited in DDBJ/EBI/GenBank database by using blastp search with *E. histolytica* G3PDH and GK, described in this paper, as a query. Multiple sequence alignment of these proteins was generated using CLUSTAL W program^48^. For identification and structural modeling, amino acid sequence of the putative *E. histolytica* glycerol-3-phosphate dehydrogenase (EhG3PDH) was subjected to PSI BLAST search using the Phyre2 protein recognition server (http://www.sbg.bio.ic.ac.uk/~phyre2/html/page.cgi?id=index)^14^. The identified most similar templates based on the confidence values and percent identity of the aligned coverages were obtained, and their amino acid sequences were obtained from their PDB coordinate data with which their sequence alignment were generated using ESPript 3.0 alignment editor^16^. The predicted/modelled three-dimensional structure was visualized and processed with Pymol program^49^. The presence of the conserved core signatures for members the sugar kinases superfamily (*i.e.* βββαβαβα structure motif) was investigated by protein sequence multiple alignment. Sequence of GKs from different organisms—*Enterococcus casseliflavus*, *Staphylococcus aureus*, *Escherichia coli, T. b. gambiense*, *Homo sapiens*, *Arabidopsis thaliana, and Plasmodium falciparum*, were obtained from NCBI database (https://www.ncbi.nlm.nih.gov/protein) and aligned with the sequence of *E. histolytica* GK using ESPript 3.0 alignment editor^16^. The predicted secondary structure elements and substrate binding amino acids were determined and annotated.

### Construction of a plasmid for the production of recombinant *E. histolytica* G3PDH and GK

Standard techniques were used for cloning and plasmid construction as previously described^50^. A fragment was cloned to produce a fusion protein containing a histidine-tag (provided by the vector) at the amino terminus. A DNA fragment corresponding to cDNA encoding *E. histolytica* G3PDH and GK was amplified by PCR using the *E. histolytica* cDNA library^17^ as a template and oligonucleotide primers. The sense and antisense oligonucleotide primers used for amplification of the gene for EhG3PDH were: 5′-GAA**GGATCC**ATGTCTTCTTATGATATAGTC-3′; 5′-GAA**GTCGAC**TTATTCTTCTAAAGCAACAAC-3′; and those for *EhGK* gene were: 5′-GAC**GGATCC**ATGAAATATATCCTTGCTATT-3′ and 5′-GAA**GTCGAC**CTAATGGACAAATGAACGTGC-3′, where bold letters indicate *Bam*HI and *Sal*I restriction sites. PCR was performed with platinum *pfx* DNA polymerase (Invitrogen) and the following parameters: an initial incubation at 94 °C for 2 min; followed by the 30 cycles of denaturation at 94 °C for 15 s; annealing at 50, 45, or 55 °C for 30 s; and elongation at 68 °C for 2 min; and a final extension at 68 °C for 10 min. The PCR fragment was digested with *Bam*HI and *Sal*I, electrophoresed, purified with Gene clean kit II (BIO 101, Vista, CA, USA), and ligated into *Bam*HI and *Sal*I digested pCOLD-1 (Novagen) in the same orientation as the T7 promoter to produce pCOLD1-EhG3PDH and pCOLD1-EhGK. The nucleotide sequences of the cloned EhG3PDH and EhGK were verified by sequencing to be identical to the putative protein coding regions of EhG3PDH (EHI_099700 and XM_644519, XP_649611) and the EhGK gene (EHI_164850 and XM_650121, XP_655213).

### Bacterial expression and purification of recombinant *E. histolytica* G3PDH (rEhG3PDH) and GK (rEhGK)

The above mentioned plasmids was introduced into *E. coli* BL21 (DE3) cells by heat shock at 42 °C for 1 min. *E. coli* BL21 (DE3) harboring pCOLD1-EhG3PDH and pCOLD1-EhGK was grown at 37 °C in 100 ml of Luria Bertani medium in the presence of 50 µg/ml ampicillin. The overnight culture was used to inoculate 500 ml of fresh medium, and the culture was further continued at 37 °C with shaking at 180 rpm. When A_600_ reached 0.6, 1 mM of isopropyl β-d-thio galactopyranoside was added, and cultivation was continued for another 24 h at 15 °C. *E. coli* cells from the induced culture were harvested by centrifugation at 4050×*g* for 20 min at 4 °C. The cell pellet was washed with PBS, pH 7.4, re-suspended in 20 ml of the lysis buffer (50 mM Tris–HCl, pH 8.0, 300 mM NaCl, and 10 mM imidazole) containing 0.1% Triton X100 (v/v), 100 µg/ml lysozyme, and 1 mM phenylmethyl sulfonyl fluoride, and incubated at room temperature for 30 min, sonicated on ice and centrifuged at 25,000×*g* for 15 min at 4 °C. The supernatant was mixed with 1.2 ml of 50% Ni^2+^-NTA His-bind slurry, incubated for 1 h at 4 °C with mild shaking. The resin in a column was washed three times with buffer A [50 mM Tris–HCl, pH 8.0, 300 mM NaCl, and 0.1% Triton X-100, v/v] containing 10–50 mM of imidazole. Bound proteins were eluted with buffer A containing 100–300 mM imidazole. After the integrity and the purity of recombinants protein were confirmed with 12% SDS-PAGE analysis, followed by Coomassie Brilliant Blue staining, they were extensively dialyzed twice against the 300-fold volume of 50 mM Tris–HCl, 150 mM NaCl, pH 8.0 and the cOmplete Mini protease inhibitor cocktail (Roche, Mannheim, Germany) for 18 h at 4 °C. The dialyzed proteins were stored at − 80 °C with 20% glycerol in small aliquots until further use. Protein concentrations were spectrophotometrically determined by the Bradford method using bovine serum albumin as a standard as previously described^51^. The recombinant EhGK was overproduced at the level of 20–25% of the total soluble proteins in *E. coli* BL21 cells. SDS-PAGE analysis followed by Coomassie Brilliant Blue staining showed that the purified rEhGK protein was present as a single 56.3-kDa homogenous protein, under reducing conditions (Supplementary Fig. S1B). The mobility of rEhGK was consistent with the predicted size of the monomeric EhGK proteins with an extra 2.6 kDa histidine tag added at the amino terminus. The purity of rEhGK was estimated more than 95% as judged by densitometric scanning of the stained gel. rEhGK protein was stable and retained their full activity when stored with 10–15% glycerol at − 30 or − 80 °C for at least 3 months. Recombinant EhG3PDH was expressed in *E. coli* BL21 (DE3) mainly as insoluble inclusion bodies (Supplementary Fig. S1A), and thus enzymological studies for G3PDH were not conducted.

### Enzyme assays

Kinetic studies of EhGK for both the forward and reverse reactions, initial-velocity determinations were carried out over wide concentration ranges. The data obtained were fitted for the Michaelis–Menten plot and transformed to double-reciprocal plots (Fig. 5A–D) for estimation of kinetic constants, *K*_m_ and *V*_max_. The forward glycerol kinase (glycerol + ATP → G3P + ADP) activity was monitored in a coupling assay as previously described^52^, in which resazurin reduction was monitored. The fluorescence intensities were continuously measured to estimate the formation of resorufin at 37 °C by excitation at 530 nm and emission at 590 nm in a reaction mixture comprising 2 mM Glycerol, 2 mM ATP, 50 mM Tris HCl pH 7.5, 5 mM MgCl_2_, 0.01% triton, 0.005% BSA, 1 mM Glucose, 0.1 mM NADP^+^, 0.05 mM resazurin, 10 mM N-ethylmaleimide, 1 unit each of ADP hexokinase, G6P dehydrogenase and diaphorase and 2 µg of recombinant GK. The kinetic parameters, *K*_m_ and *V*_max_ for ATP were determined at 2 mM constant concentration of glycerol, while varying the concentration of ATP from 0.01 to 3 mM; the parameters were determined for glycerol at 2 mM constant concentration of ATP over a concentration range of 0.002–3 mM of glycerol.

The reverse glycerol kinase (G3P + ADP → glycerol + ATP) activity was monitored in a coupling assay as previously described^53^ in which production of NADPH was monitored. The reaction mixture composed of 50 mM Tris HCl pH 7.5, 1 mM EDTA, 5 mM MgSO_4_, 0.5 mM NADP^+^, 50 mM glucose, 2 mM ADP, 10 mM G3P and 1 unit each of hexokinase and glucose-6-phosphate dehydrogenase and 2 µg of recombinant GK. The ATP produced by EhGK is utilized by hexokinase to generate glucose 6-phosphate from glucose, and finally glucose-6-phosphate dehydrogenase catalyses the production of NADPH from glucose 6-phosphate and NADP^+^. The rate of NADPH accumulation was measured spectrophotometrically at 340 nm using the JASCO V-660 spectrophotometer. The kinetic parameters, *K*_m_ and *V*_max_ for ADP were determined at 40 mM constant concentration of G3P, while varying the concentration of ADP from 0.025 to 1.2 mM; the parameters were determined for G3P at 1 mM constant concentration of ADP over a concentration range of 0.8–40 mM of G3P. Kinetic data were estimated by curve fitting with the Michaelis–Menten equation using GraphPad Prism (GraphPad Software Inc., San Diego, USA). The experiment was repeated three times in triplicate with proteins isolated from two independent extractions, and kinetic values are presented as the means ± S.E. for three independent kinetic assays.

### Production of *E. histolytica* transformants overexpressing EhG3PDH and EhGK

The protein coding region of EhG3PDH and EhGK was amplified by PCR from cDNA using sense and antisense oligonucleotides containing appropriate restriction sites at the 5′ end. The sense and antisense oligonucleotide primers used for EhG3PDH were: 5′-CTA**CCCGGG**ATGTCTTCTTATGATATAGTC-3′; 5′-CGA**CTCGAG** TTATTCTTCTAAAGCAACAAC-3′ and for EhGK were: 5′-CTA**CCCGGG**ATGAAATATATCCTTGCTATT-3′; 5′-AAT**CTCGAG**CTAATGGACAAATGAACGTGC-3′ (restriction sites are shown in bold). The PCR-amplified DNA fragment was digested with *Sma*I and *Xho*I, and ligated into *Sma*I and *Xho*I sites of the expression vector pEhExHA^54^ to produce pEhExHA-G3PDH and pEhExHA-GK. Plasmids were introduced into the amoeba trophozoites by liposome-mediated transfection as previously described^55^. Transformants were initially selected in the presence of 3 µg/ml of geneticin. The geneticin concentration was gradually increased to 6–20 µg/ml during next 2 weeks before the transformants were subjected to analyses.

### Production of *EhG3PDH* and *EhGK* gene-silenced strain

In order to construct a plasmid for epigenetic silencing^19^ of *EhG3PDH* and *EhGK*, a fragment corresponding to a 420-bp long 5′ end of open reading frame of *EhG3PDH and EhGK* gene was amplified by PCR from cDNA using sense 5′-GAG**AGGCCT**ATGTCTTCTTATGATATAGTC-3′ and antisense 5′-GAT**GAGCT**CAATAGCTTTCTTGATATTTTC-3′ for EhG3PDH and 5′-GAG**AGGCC**TATGAAATATATCCTTGCTATT-3′ and 5′-GAT**GAGCTC**CATAATTTTACTAGCACTGAA-3′ for EhGK. (restriction sites are shown in bold). The PCR amplified products were digested with *Stu*I and *Sac*I, and ligated into the *Stu*I- and *Sac*I-double digested pSAP2-Gunma to construct gene silencing plasmids pSAP2G-G3PDH and pSAP2G-GK. The trophozoites of G3 strain were transformed with either empty vector or silencing plasmid by liposome-mediated transfection as previously described^56^. Transformants were initially selected in the presence of 1 μg/ml geneticin, and the geneticin concentrations were gradually increased during next 2 weeks prior to subjecting the transformants to analyses.

### Growth assay of *E. histolytica* trophozoites

Approximately 6 × 10^6^ exponentially growing trophozoites of *E. histolytica* G3 strain transformed with pSAP2G-G3PDH, pSAP2G-GK, and pSAP2G (control) were inoculated into 6 ml of fresh BI-S-33 medium containing 10 μg/mL geneticin, and the parasites were counted every 24 h on a haemocytometer.

### Glycerol quantitation

To estimate the amount of glycerol approximately 10^7^ normally cultured trophozoites were quickly harvested by centrifugation at 500×*g* for 5 min. The cellular pellets were extracted with perchloric acid, as described earlier^13^ and used for the estimation of intracellular glycerol. Glycerol concentration was determined by using glycerol estimation kits from Biovision (Mountain View, CA).

### Hydrogen peroxide (H_2_O_2_) sensitivity assay

To examine sensitivity to H_2_O_2_, *E*. *histolytica* G3PDH, GK gene-silenced and control (harboring plasmid pSAP2G) transformants were cultured in BI-S-33 media containing 10 μg/mL geneticin for 48 h at 35.5 °C. After 48 h, approximately 10,000 trophozoites per well were seeded into the wells of a 96-well plate containing BI-S-33 medium supplemented with 10 μg/mL geneticin and further incubated for 1 h at 35.5 °C. The trophozoites were then exposed to H_2_O_2_ (0, 0.8, 1.6, 2.4, 3.2, 4, 4.8 and 6.4 mM) for 1 h. After incubation, the medium was removed and 90 μl of pre-warmed Opti-MEM I (Life Technologies) and 10 μl WST-1 solution (Roche Diagnostics, Mannheim, Germany) were added to each well. Viability of trophozoites was detected by measuring absorbance at 450 nm using a microplate reader (Spectramax paradigm multimode plate reader). The sensitivity assays were performed in triplicate and repeated at least three times.

### RNA-seq analysis

*E. histolytica* trophozoites were harvested form logarithmic growth phase of pSAP2G (mock transfected), GK and G3PDH gene silenced strains. Total RNA was purified using TRIZOL reagent (Thermo Fisher, Waltham, MA, USA) according to manufacturer’s instruction. Total RNA samples from three biological replicates were sent for RNA sequencing (Macrogen, Kyoto, Japan). RNA-seq libraries were generated by using TruSeq stranded mRNA kit. The protocol included polyA plus RNA purification, RNA fragmentation, random hexamer primed cDNA synthesis, linker ligation, PCR amplification, and gel purification. The libraries were then subjected to 100nt paired-end sequencing using an Illumina (NovaSeq6000) (Illumina, San Diego, CA, USA). Raw image files were processed using the Illumina  Real Time Analysis (RTA) software^57^. Averaged 6,084,785,041 total bases and 60,245,396 total reads was obtained. Data analysis was conducted by Tohoku Kagaku. Briefly, adaptor sequences were removed then low quality reads were removed. Obtained high quality reads were mapped to *E. histolytica* template genome then Fragments Per Kilobase of exon per Million mapped fragments (FPKM) were calculated and used for expression value (Tohoku kagaku, Iwate, Japan).

### Cell fractionation and immunoblot analysis

Trophozoites of the ameba transformant expressing pEhExHA-G3PDH and pEhExHA-GK, were washed three times with PBS containing 2% glucose. After resuspension in homogenization buffer (50 mM Tris–HCl, pH 7.5, 250 mM sucrose, 50 mM NaCl and 0.5 mg/ml E-64 protease inhibitor), the cells were disrupted mechanically by a Dounce homogenizer on ice, centrifuged at 500×*g* for 5 min, and the supernatant was collected to remove unbroken cells. The supernatant fraction was centrifuged at 5000×*g* for 10 min to isolate pellet and supernatant fractions. The 5000×*g* supernatant fraction was further centrifuged at 100,000×*g* for 60 min to produce a 100,000×*g* supernatant and pellet fractions. The pellets at each step were further washed twice with homogenization buffer and re-centrifuged at 100,000×*g* for 10 min to minimize carryover.

Cell lysates and culture supernatants were separated on 12–15% (*w/v*) SDS-PAGE and subsequently electro transferred onto nitrocellulose membranes (Hybond-C Extra; Amersham Biosciences UK, Little Chalfont, Bucks, UK) as previously described^58^. Non-specific binding was blocked by incubating the membranes for 1.5 h at room temperature in 5% non-fat dried milk in TBST (50 mM Tris–HCl, pH 8.0, 150 mM NaCl and 0.05% Tween-20). The blots were reacted with anti-HA mouse monoclonal (clone 11MO, Covance, Princeton, NJ, USA), anti-cysteine protease binding family protein 1 (CPBF1)^18^, and anti-cysteine synthase 1 (CS1) rabbit polyclonal antisera^17^ at a dilution of 1:500 to 1:1000. CPBF1 and CS1 served as controls for membrane and cytosolic fractions, respectively. The membranes were washed with TBST and further reacted either with horse radish peroxidase-conjugated anti-mouse or anti-rabbit IgG antisera (1:20,000) (Invitrogen) at room temperature for 1 h. After washings with TBST, specific proteins were visualized with alkaline phosphatase conjugate substrate kit (Bio-Rad) and images were scanned with Image Scanner (Amersham Pharmacia Biotech, Piscataway, NJ, USA) or the fluorescent signal of each protein was measured with a chemiluminescence detection system (Millipore) using Scion Image software (Scion Corp., Frederick, MD).

### Supplementary Information

Supplementary Information 1.

Supplementary Information 2.

Supplementary Information 3.

Supplementary Information 4.

## Data Availability

The RNA-seq data generated and/or analyzed in this study are accessible in the Gene Expression Omnibus repository under accession number GSE240436. The datasets generated during and/or analysed during the current study are available from the corresponding author on reasonable request.
