# Supplementary material for: Glycerol biosynthetic pathway plays an essential role in proliferation and antioxidative defense in the human enteric protozoan parasite Entamoeba histolytica
Source: Sci Rep. 2023 Sep 5;13:14596. doi: 10.1038/s41598-023-40670-z (PMC10480196; doi:10.1038/s41598-023-40670-z)
Supplement: Supplementary file 4 — Supplementary Information 4. [file 41598_2023_40670_MOESM4_ESM.docx]

**Glycerol biosynthetic pathway plays an essential role in** **proliferation and antioxidative defense in the human enteric protozoan parasite *Entamoeba histolytica***

**Ghulam Jeelani^1^, Emmanuel Oluwadare Balogun^1,2^, Afzal Husain^3^ and Tomoyoshi Nozaki^1^**

^1^Department of Biomedical Chemistry, Graduate School of Medicine, The University of Tokyo, Japan.

^2^Department of Biochemistry, Ahmadu Bello University, Zaria, Nigeria.
^3^Department of Biochemistry, Faculty of Life Sciences, Aligarh Muslim University, Aligarh, India.

**Supplementary Fig. S1**

**The protein profile of each fraction during purification of recombinant EhG3PDH (A) and EhGK (B).** Protein samples at each step of purification were subjected to 5-20% (A) or 15% (B) SDS-PAGE under reducing conditions, and then stained with Coomassie Brilliant Blue R250. A protein band corresponding to the recombinant EhG3PDH (A) or EhGK (B) is indicated by red arrow.

**Supplementary Fig. S2**

**Multiple sequence alignment of the N- and C-terminal portions of G3PDH from various organisms. A)** Multiple sequence alignment of the N-terminal domain (predicted as G3PDH) of G3PDHs from *E. histolytica* and other species. The species name abbreviations and the NCBI accession numbers (in parenthesis) are as follow: *Entamoeba histolytica* (XP_649611), *Giardia lamblia* (XP_001707988), *Escherichia coli* anaerobic-glycerol-3-phosphate dehydrogenase GlpA (WP_060643474), *Escherichia coli* aerobic glycerol-3-phosphate dehydrogenase GlpD (EED1438795), *Homo sapiens* mitochondrial glycerol-3-phosphate dehydrogenase (NP_000399). A white unshaded box indicates flavin adenine dinucleotide (FAD)-binding motif and a grey box depicts a stretch of amino acid residues potentially involved in G3P binding (Yeh, et al, 2008). The conserved residues are marked by asterisks (*) while similar amino acids are shown with either periods (.) or colons (:). Sequence alignment was performed using Clustal Omega (Sievers et al 2018). **B)** Multiple sequence alignment of the C-terminal domain (predicted as disulfide/persulfide reductase) of EhG3PDH with the orthologs from *Giardia lamblia,* disulfide reductase from *Enterococcus faecalis*, NADH persulfide reductase from *Shewanella loihica* and CoA disulfide reductase from *Bacillus anthracis*,*.* The GenBank accession numbers for the enzymes are as follows: *Enterococcus faecalis* (WP_033597645), *Shewanella loihica* (WP_011864535), *Bacillus anthracis* (WP_001110220). A white unshaded box indicates flavin adenine dinucleotide (FAD)-binding motif and a grey box depicts the residues potentially involved in NADH/NAD(P)H binding region. The presence of glycine at the last position of the motif GXGXX(G/A/P) (residues 679 –684 of EhG3PDH) and the conserved glutamic acid (703) in the NADH/NAD(P)H binding domain suggest that these enzymes prefer NADH to NADPH as a cofactor.

**Supplementary Fig. S3**

**Cellular distribution of EhG3PDH and EhGK in *E. histolytica* trophozites.** Trophozoites of the transformant strains expressing HA-tagged EhG3PDH or EhGK were fractionated as described in Materials in Methods, and subjected to immunoblot analysis using anti-HA antibody, anti-CPBF1, and anti-CS1 antisera. CPBF1 and CS1 served as control of organelle and cytosolic markers, respectively. The full image of immunoblots are shown in Supplementary Fig. S6

**Supplementary Fig. S4**

**TLC chromatogram showing both polar and non-polar lipids.** Red boxes indicate cropped regions presented in Figure 2B.

**Supplementary Fig. S5**

**Confirmation of transcriptional silencing of EhG3PDH and EhGK.** Red boxes indicate cropped regions presented in Figure 6A.

**Supplementary Fig. S6**

**Cellular fractionation and immunoblot analysis of** **EhG3PDH and EhGK**. Red boxes indicate cropped regions of the immunoblots presented in Figure S3
